# Unexpected Terrain Induced Changes in Cortical Activity in Bipedal-Walking Rats

**DOI:** 10.3390/biology11010036

**Published:** 2021-12-27

**Authors:** Honghao Liu, Bo Li, Minjian Zhang, Chuankai Dai, Pengcheng Xi, Yafei Liu, Qiang Huang, Jiping He, Yiran Lang, Rongyu Tang

**Affiliations:** 1 School of Mechatronical Engineering, Beijing Institute of Technology, Beijing 100081, China; 3120205085@bit.edu.cn (H.L.); 3120170125@bit.edu.cn (B.L.); 3120170166@bit.edu.cn (M.Z.); daichuankai5713@sina.cn (C.D.); 3120185110@bit.edu.cn (P.X.); yafei.liu@bit.edu.cn (Y.L.); qhuang@bit.edu.cn (Q.H.); jiping.he@bit.edu.cn (J.H.); 2 Beijing Innovation Centre for Intelligent Robots and Systems, Beijing Institute of Technology, Beijing 100081, China

**Keywords:** EEG, kinematics, unexpected terrains, treadmill, bipedal-walking rats

## Abstract

**Simple Summary:**

Most studies on cortical dynamics during walking require subjects to walk stably on specific terrain. In fact, humans or other animals are often disturbed by an abrupt change in terrains during walking. To study the impact of unexpected terrain on cortical activity, we analyzed the kinematics and electroencephalography (EEG) dynamics of bipedal-walking rats after encountering unexpected terrain. We found that the gait of rats after encountering the unexpected terrain were significantly different from normal walking. Furthermore, the activities of the left and right primary motor areas (M1), the left and right primary somatosensory areas (S1), and the retrosplenial area (RSP) are coupled to gait cycle phase and varied with the terrain conditions. These findings suggest that unexpected terrains induced changes in gait and cortical activity, and provide novel insights into cortical dynamics during walking.

**Abstract:**

Humans and other animals can quickly respond to unexpected terrains during walking, but little is known about the cortical dynamics in this process. To study the impact of unexpected terrains on brain activity, we allowed rats with blocked vision to walk on a treadmill in a bipedal posture and then walk on an uneven area at a random position on the treadmill belt. Whole brain EEG signals and hind limb kinematics of bipedal-walking rats were recorded. After encountering unexpected terrain, the θ band power of the bilateral M1, the γ band power of the left S1, and the θ to γ band power of the RSP significantly decreased compared with normal walking. Furthermore, when the rats left uneven terrain, the β band power of the bilateral M1 and the α band power of the right M1 decreased, while the γ band power of the left M1 significantly increased compared with normal walking. Compared with the flat terrain, the θ to low β (3–20 Hz) band power of the bilateral S1 increased after the rats contacted the uneven terrain and then decreased in the single- or double- support phase. These results support the hypothesis that unexpected terrains induced changes in cortical activity.

## 1. Introduction

When humans and other animals walk, they cannot detect terrain changes in time due to blocked vision such as in low-light environment. Previous studies have reported that humans and animals can adjust their gait to adapt to unexpected terrains. For example, when humans encounter an unexpected slip during walking, they will choose an appropriate strategy to restore stability based on previous experience [1]. In addition, humans will consciously maintain posture stability by increasing step time and step width when walking on uneven terrains, especially in low-light environments [2]. Studies have demonstrated that the guinea fowl can also quickly adjust its gait to prevent falling when it encounters a sudden height decrease in the terrain [3], and these adjustments are thought to be related to the spinal cord [4,5,6]. Furthermore, walking is thought to be driven by the spinal cord and subcortical neural circuits, and rarely requires the involvement of the cortex [7,8,9]. As such, these studies show that the brain contributes little to responding to and overcoming unexpected terrains during walking.

Recent studies have reported that the cortex is actively involved in walking in humans and animals [10,11,12,13,14]. Gwin et al. found that electrocortical activity is coupled with the gait cycle phase when humans walk steadily on a treadmill [15]. In addition, there is a significant difference in electrocortical activity in humans between passive walking with the assistance of robots and active walking [16]. Furthermore, a previous study has demonstrated that the brain areas related to sensory processing and integration are more involved in walking when human vision cannot be used for locomotor guidance, and there are also higher requirements for neural processing related to motor planning and execution [17]. It is worth noting that cortex activity seems to be affected by task difficulty. For example, compared with normal walking, electrocortical activity in humans changes significantly when walking on slopes [18]. Cats can also actively adjust their limbs to negotiate the obstacles on treadmill, and the motor cortex and posterior parietal cortex play an important role in this process [19,20]. A previous study on rodents has demonstrated that the motor cortex actively participates in the movement control of the hind limbs when rats walk freely on flat ground, a treadmill, or stairs [21] and the brain connectivity showed different changes when rats walk on slopes with different inclination angles [22]. In addition, the sensorimotor cortex of rats actively involved in the adjustment of gait during bipedal walking on a treadmill or stairs [23].

Several studies have focused on the impact of unexpected external disturbances on cortical dynamics [24,25,26,27]. One study has reported that the premotor cortex and supplementary motor area actively participate in navigating unpredictable obstacles when humans walk or run on a treadmill, and posterior parietal cortex activity changed with locomotion speed [28]. An electrocortical study has demonstrated that the theta band power of the anterior cingulate, anterior parietal, and superior dorsolateral-prefrontal increases significantly when humans lose their balance during walking on a balance beam [29]. When the posture stability is disturbed by the sudden acceleration of the support plane, humans can quickly respond according to the requirements, and the power of multiple brain areas, such as the anterior prefrontal cortex changes significantly [30]. Studies have also reported that animals can adjust their limbs to maintain body stability when the walking or standing plane inclined laterally, and the motor cortex is activated in this process [31,32]. 

Until now, too little attention has been paid to the impact of unexpected terrains, especially uneven terrains, on cortical activity during walking. To address this question, we used rats as the model and built a movement assistance platform to help the rats walk on the treadmill in a bipedal posture. In addition, we attached an uneven area on the treadmill belt and blocked the rats’ sight to remove all visual cues on the upcoming change in the terrain. We built an invasive multi-modal recording platform to record the electroencephalography (EEG) signals from the whole cortex and the kinematics of the hind limbs. Subsequently, we used independent component analysis (ICA) to decompose the EEG signals into the multiple independent sources and to calculate the equivalent current dipole for each independent component. The dipoles were clustered according to the k-mean algorithm, and the average event-related spectral perturbation (ERSP) and power spectral density (PSD) of each cluster was analyzed. We found that after the rats encountered the unexpected terrain, their gait and cortical activity were significantly different from normal walking, and varied with the terrain conditions.

## 2. Materials and Methods

### 2.1. Animals

The experiments were conducted on six male Sprague Dawley (SD) rats [33] weighing 250–300 g. SD rats were widely used in behavior and neuroscience studies due to their docile nature, strong adaptability, and excellent reproductive performance [34,35,36,37]. Each rat was individually maintained in a plexiglass cage with 12 h light/12 h dark cycles. The temperature, humidity, and ventilation conditions in the cage were appropriate, and food and water were provided. Rats were 9–11 weeks old when the experiments commenced. SD rats in this period were mature and have perfect motor function [38], making them ideal for our study. All animal procedures were approved by the Institutional Animal Care and Use Committee of Beijing Institute of Technology.

### 2.2. Experimental Design

**Figure 1:**
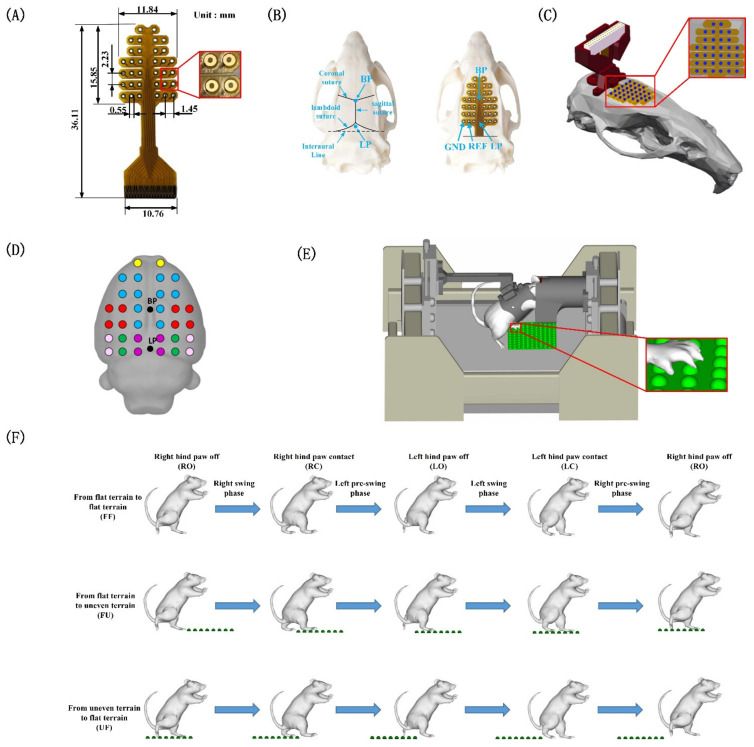
Experimental setup. (**A**) The 32-channel flexible electrode arrays used in this study. (**B**) The installation position of the electrode on the rat skull and electrode locations were based on stereotaxic coordinates from the bregma point (BP) and lambda point (LP). The BP is defined as the intersection of the coronal and sagittal sutures. LP (midpoint of the curve of best fit along the lambdoid suture) is 0.3 mm anterior to the coronal plane passing through the interaural line. (**C**) The electrodes were fixed on the skull with screws and a protective shell was installed. The blue dots represent screws that attach the electrodes array to the skull. (**D**) The putative electrode positions on the rat’s brain surface, as determined by Brainstorm3. The colors of the electrodes correspond to the different brain regions (yellow: frontal area; blue: somatomotor area; red: somatosensory area; purple: retrosplenial area; green: visual area; pink: posterior parietal association area). (**E**) The rat walked in a bipedal posture on the treadmill with the help of a suspension device, and a black tube was used to block all frontal visual cues on the upcoming terrain. A removable uneven area was set on the surface of the treadmill belt. (**F**) Behavioral tasks, gait events, and gait cycle phases were analyzed.

As shown in Figure 1E, each rat was suspended with a jacket that worn on the upper body and allowed to walk on a moving treadmill belt (6.5 cm/s–7.5 cm/s) in a bipedal posture. A removable uneven area (11 cm × 11 cm) was set on treadmill belt and hemispherical protrusions with a diameter of 8 mm were unevenly distributed inside of the uneven area. A total of four cameras (80 Hz) were placed on the left and right sides of the rat to record the behavior. During the whole process, the head of the rat was covered by a black tube to block all frontal visual cues on the upcoming terrain. First, we trained each rat to walk on the treadmill in a bipedal posture (without uneven areas) for two weeks to adapt the animals to bipedal walking. Each rat trained for 60 min a day, and each trail lasted for 5 min, followed by rest for 5 min (a food reward was given after each trail). In the formal experiment, the uneven area was set at a random position on the treadmill belt and moved along with the belt. After the rat encountered and successfully passed the uneven area, we turned off the treadmill and adjusted the position of the uneven area on the belt to ensure that the uneven area appeared randomly. The rats rested for 5 min after repeating the above process ten times. Each rat completed 50–60 trials on average a day. It is worth noting that the length of the uneven area was not enough to support the rat to walk continuously. After the rat completely transitioned to the uneven area, it could leave the area in the next step. In this study, two rats were excluded due to hardware deficiencies and infection issues, and data from four rats were used for subsequent analysis.

As shown in Figure 1F, three behavioral processes were designed into the experimental course as follows: (1) the rat walked from the flat terrain to the flat terrain (FF); (2) the rat walked from the flat terrain to the uneven terrain (FU); and (3) the rat walked from the uneven terrain to the flat terrain (UF). We selected the complete gait cycle in the three behavioral tasks for analysis. Each gait cycle included five events as follows: right hind paw coming off the ground (RO), right hind paw contacting the ground (RC), left hind paw coming off the ground (LO), left hind paw contacting the ground (LC) and right hind paw coming off the ground (RO). Each gait cycle started when the rat’s right hind paw left the ground and ended when the right hind paw left the ground again, and the complete gait cycle was divided into four phases as follows: (1) from the right hind paw coming off the ground to the right hind paw contacting the ground (right swing phase); (2) from the right hind paw contacting the ground to the left hind paw coming off the ground (left pre-swing phase); (3) from the left hind paw coming off the ground to the left hind paw contacting the ground (left swing phase); and (4) from the left hind paw contacting the ground to the right hind paw coming off the ground (right pre-swing phase). The four phases under the three experimental conditions were analyzed by Simi Motion v9.2.2 (Simi Inc., Unterschlessheim, Germany). In this study, three metrics were used to assess the gait features: (1) time interval of each phase; (2) gait length (displacement of the hind paw in the horizontal direction) of the left swing phase and right swing phase; (3) locomotion velocity (swing velocity of the hind limbs) of the left swing phase and right swing phase. These metrics were selected because they reflect the differences in the behavior of rats during terrain transition. We obtained the time of each gait event by visually observing the motion video, which was used to calculate the time interval of each phase and to mark the gait events in the EEG data. The gait length and locomotion velocity were obtained by using the kinematics analysis function in Simi Motion. One-way ANOVA was used to compare the time interval, gait length, and locomotion velocity of each gait phase under the different experimental conditions.

### 2.3. Surgical Procedures

All surgical procedures were performed in a sterile environment, and rats were anesthetized with 2–3% isoflurane. The skin on the head was incised to expose the skull and washed with 2.5–3.5% hydrogen peroxide. The electrode array (Figure 1A) was attached to the surface of the skull with 34 screws. Figure 1B shows the position of the electrode array on the skull. The screws penetrate the skull but did not penetrated the dura mater. Figure 1D shows the projection position of the electrode array on the brain surface. An insulating shell was placed on the electrode interface to protect the exposed area (Figure 1C). The shell was also fixed on the surface of the skull with screws. The electrode array was covered with dental cement to prevent infection and protect the hardware. The incision was disinfected and sutured. After surgery, the rats were maintained in their cages for one week for recovery.

### 2.4. Data Acquisition

In this study, we recorded the kinematics and EEG data of rats. The kinematics data were recorded by four cameras (80 Hz) using the Plexon CinePlex system (Plexon Inc., Dallas, TX, USA). The motion of each rat was recorded and saved synchronously with EEG data for behavioral analysis and gait event extraction. The EEG data were collected by the home-designed 32-channel electrode array (Figure 1A). The EEG electrodes array was fabricated on a flexible polyimide substrate using photolithography, metal deposition, and etching techniques. The array was comprised of 34 microelectrodes, each with a diameter of 500 µm, which include 32 working electrodes, one reference electrode (REF), and one ground electrode (GND). The GND and the REF were both on the same side and were not symmetrical (Figure 1B). It is worth noting that the signals from the GND and the REF were excluded, and only the signals from the 32 working electrodes were retained for analysis. The interface of the electrode array was connected to the Digital Headstage processor (Plexon Inc.) through a cable. The EEG data were sampled at 2000 Hz and a notch filter was used to reduce line noise (50 Hz). The EEG data were transmitted to the OmniPlex neural data acquisition system (Plexon Inc.) for storage.

### 2.5. EEG Analysis

**Figure 2:**
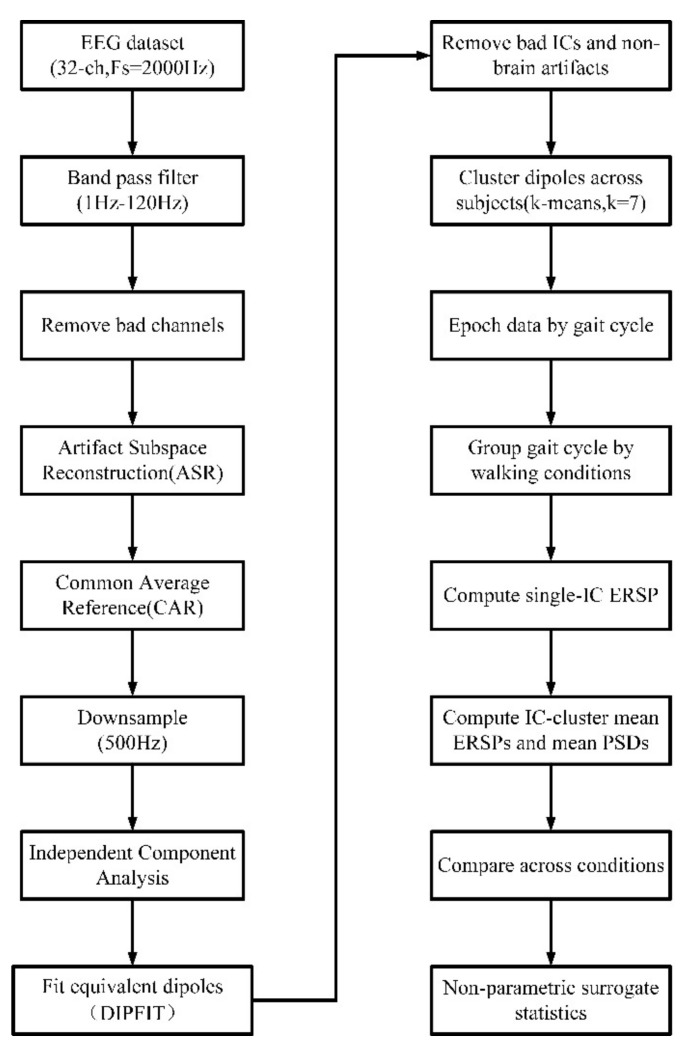
Flow-chart of the EEG processing pipeline.

EEG data analysis was performed using custom scripts written in MATLAB v2018b (The MathWorks, Inc., Nedick, MA, USA) containing functions from EEGLAB [39]. The EEG signal processing methodology is shown in Figure 2. First, the EEG data were filtered between 1 Hz and 120 Hz (zero phase FIR filter, order 220). EEG channels that were flat for more than 5 s or at kurtosis of more than five standard deviations from the mean were removed. An average of 31 channels were retained for each subject. The Artifact Subspace Reconstruction (ASR) algorithm in EEGLAB was applied to remove high-amplitude artifacts [40]. The EEGLAB function ‘clean_windows’ was used to automatically select clean data segments from the recorded data as the calibration data for ASR. In this study, a 500-ms sliding window and a variance threshold of three standard deviations were used to identify corrupted subspaces. The EEG data were re-referenced to the common average and down-sampled to 500 Hz. Subsequently, the EEG data were decomposed into maximally independent components (ICs) by Infomax independent component analysis. The DIPFIT toolbox in EEGLAB was used to estimate the location of cortical sources of ICs [39]. We selected a standard head model in the DIPFIT toolbox and manually adjust the electrode position to fit it. Next, we calculated the equivalent current dipole to fit the scalp projection of each IC source. This procedure resulted in dipole locations inside a standard head model. We removed the ICs whose equivalent current dipoles were located outside the head model, and selected those in which the equivalent dipoles explained >85% of the variance of the IC scalp projections for analysis [18]. In addition, we also removed those ICs related to non-brain artifacts (for example shaking head or noises), by visually inspecting each IC scalp projection and power spectra. After the above processing, an average of 13 brain related ICs per rat were retained for further analyses. Next, the k-means algorithm was used to cluster the ICs from all subjects. The k-means clustering algorithm is an iterative clustering analysis algorithm [41]. Specifically, we set a seven-cluster centroid (*k* = 7) and assign each IC to the cluster centroid closest to it according to IC scalp projection, power spectra, and dipole locations. The ICs that exceeded the three standard deviations of the cluster centroid were removed. Finally, the ICs from all rats were clustered into seven clusters located in the head model. It is worth noting that the ICs in some clusters were came from less than half of the rats, and these clusters were excluded. We extracted epochs from the EEG data based on the rat’s hind limb kinematics data (453 epochs in total). Each epoch started 500 ms before the event RO and the total duration was 2.5 s. The 500 ms before the event RO served as the baseline. Thereafter, we grouped these epochs according to the different walking conditions and obtained three experimental conditions as follows: FF, FU, and UF (Figure 1F). The ERSP of single IC and IC-cluster were calculated. Here, the ERSP can be viewed as a generalization of the event-related desynchronization/synchronization (ERD/ERS), which revealed the power of EEG signals in different frequency bands changes with gait events. Increased (or synchronized) power within a frequency band following the presentation of an event was defined as an ERS, and ERD refers to a decrease (or desynchronized) of power within a frequency band following an event [42]. In this study, we used the following method to calculate ERSP. First, single epoch power spectrograms were computed and time-warped to the median latency (across subjects) using linear interpolation [15]. Next, we averaged the log power spectrograms over the entire gait cycle to obtain the single IC ERSP and then averaged the values to obtain the IC-cluster mean ERSP. The non-parametric bootstrapping technique within EEGLAB was used to identify the significance of the ERSPs (*p* < 0.05). To visualize the relative timing of the spectral power fluctuations, we computed the average gait ERSP for each walking condition in the specific frequency bands of each cluster. PSD was an effective method to evaluate the distribution of signal power in the frequency domain [43]. To study the power distribution of EEG signal in different frequency bands, we calculated the average PSD of each cluster using Welch’s method and analyzed the PSD data using one-way ANOVA.

## 3. Results

### 3.1. Behavioral Analysis

**Figure 3:**
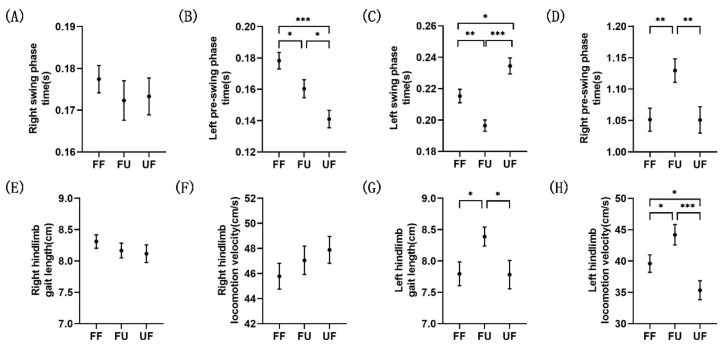
Rat hind limb motion patterns. (**A**–**D**) The mean time spent in the right swing phase, left pre-swing phase, left swing phase, and right pre-swing phase. (**E**,**F**) The mean gait length and mean locomotion velocity of the right hind limb in the right swing phase. (**G**,**H**) The gait length and locomotion velocity of the left hind limb in the left swing phase. All error bars indicate 1 SE. (*n* = 4 animals; * *p* < 0.05; ** *p* < 0.01; *** *p* < 0.001).

Figure 3 shows the comparison of the kinematics data of the different conditions in each gait phase, and the kinematics results are shown in Table 1. There was no significant difference in the kinematic metrics (Figure 3A,E,F) in the right swing phase. However, after the rat’s right hind paw contacted the next terrain, the behavior of the left hind limb changed significantly. In the left pre-swing phase, the time intervals of FU and UF conditions were significantly shorter than that of the FF condition, and that of the FU condition was significantly longer than that of the UF condition (Figure 3B; F(2,417) = 11.924, *p* < 0.001; post-hoc tests: FF vs. FU: *p* = 0.022, FF vs. UF: *p* < 0.001, FU vs. UF: *p* = 0.018). Interestingly, in the left swing phase, although the time interval of the FU condition was significantly shorter than that in FF condition, the time interval of the UF condition was longer than those of FF and FU conditions (Figure 3C; F(2,468) = 15.975, *p* < 0.001; post-hoc tests: FF vs. FU: *p* = 0.003, FF vs. UF: *p* = 0.013, FU vs. UF: *p* < 0.001). In addition, as shown in Figure 3G, the gait length of the FU condition was significantly longer than that under the other two conditions (F(2,164) = 3.344, *p* = 0.038; post-hoc tests: FF vs. FU: *p* = 0.045, FU vs. UF: *p* = 0.037). Similarly, there were significant differences in the locomotion velocity, with the fastest under FU conditions and the slowest under UF conditions (Figure 3H; F(2,181) = 7.303, *p* = 0.001; post-hoc tests: FF vs. FU: *p* = 0.029, FF vs. UF: *p* = 0.048, FU vs. UF: *p* < 0.001). In the last phase of the gait cycle, which was the right pre-swing phase, the time interval of the UF condition was significantly shorter than that of the FU condition, but there was no significant difference between UF and FF conditions (Figure 3D; F(2,470) = 5.229, *p* = 0.006; post-hoc tests: FF vs. FU: *p* = 0.003, FU vs. UF: *p* = 0.007).

### 3.2. Clusters of Independent Components

After clustering the ICs from all subjects using the k-mean algorithm, we got seven clusters. Two clusters were excluded because the ICs in each of them came from less than half of the subjects. Finally, five clusters were retained as follows: the left and right primary motor area (M1), the left and right primary somatosensory area (S1) and the retrosplenial area (RSP). Table 2 presents the Talairach coordinates of the cluster centroids, the number of subjects, and the sources contained in each cluster.

### 3.3. Event-Related Spectral Perturbation

**Figure 4:**
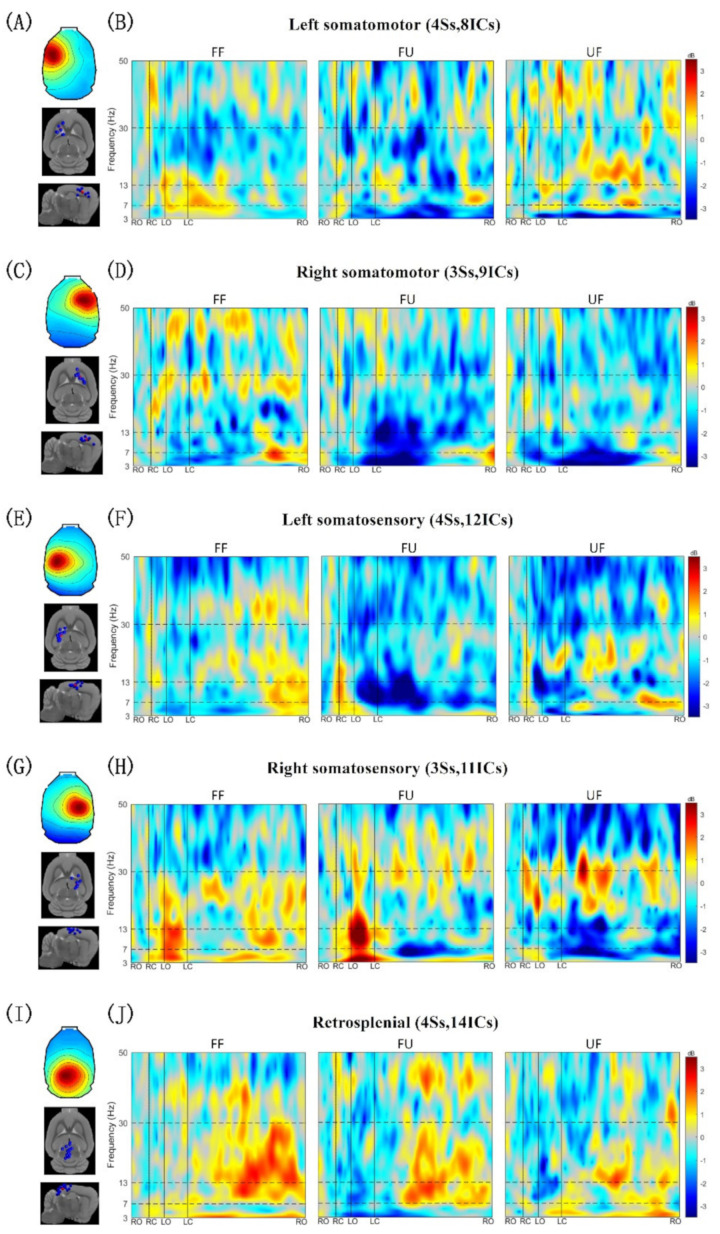
Equivalent dipole source locations, cluster mean scalp projections, and mean ERSP images for IC source clusters centered in the left and right somatomotor clusters, the left and right somatosensory clusters and the retrosplenial cluster. (**A**,**C**,**E**,**G**,**I**) Cluster mean scalp projection map and equivalent dipole locations of cluster ICs (blue spheres) and their centroid (red sphere) visualized in the MNI template brain. (**B**,**D**,**F**,**H**,**J**) Cluster mean ERSP images of rats under the different conditions (FF, FU, UF). Warm colors indicate a power increase (ERS) and the cool colors indicate a power decrease (ERD). Non-significant changes from the baseline are masked in gray (*p* > 0.05). Solid vertical lines indicate the time of the gait event.

**Figure 5:**
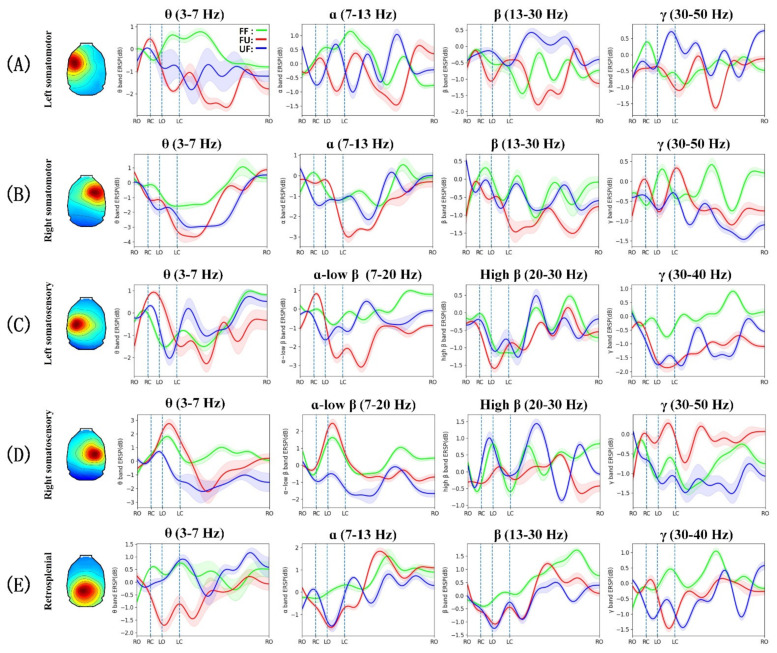
Mean ERSP for the specific frequency bands. (**A**,**B**,**E**) Mean ERSP of θ, α, β, and γ bands in the left/right somatomotor clusters and retrosplenial cluster. (**C**,**D**) Mean ERSP of θ, α to low β, high β, and γ bands in left/right somatosensory clusters. The green, red, and blue lines represent power modulations under FF, FU, and UF conditions, respectively. The 95% confidence interval envelope was plotted around the mean. Dotted vertical lines indicate the time of the gait event.

Figure 4 and Figure 5 show the gait event-related spectral perturbation of the left and right M1, the left and right S1, and the RSP under FF, FU, and UF conditions. In the left and right M1 sources (Figure 4B,D and Figure 5A,B), we found that, compared to walking on the flat terrain, a stronger ERD appeared in the θ (3–7 Hz) band of the left and right M1 under FU and UF conditions, and it spanned the entire gait cycle. When the rat’s left hind limb was ready to leaves the ground (from RC to LO), a significant ERD appeared in the α (7–13 Hz) band of the right M1, and it was strongest under the UF condition, relatively weak under the FU condition, and weakest under the FF condition. In the process of swinging the left hind limb (from LO to LC), the γ (30–50 Hz) band of the left M1 showed a significant ERS under the UF condition, while a significant ERD appeared under the other two conditions. In the last phase of the gait cycle (from LC to RO), a significant ERD appeared in the β (13–30 Hz) band of the left and right M1, and the ERD was the strongest under the FU condition.

In the left and right S1 sources (Figure 4F,H and Figure 5B,C), we noticed that from the time the right hind paw contacted the ground until the time the left hind paw left the ground (from RC to LO), a significant ERS appeared in the θ band of the left and right S1 under all conditions. In this phase, a significant ERD appeared in the α to low β band (13–20 Hz) of the right S1 under the UF condition, while a significant ERS appeared in both the left and right S1 under the other conditions. In the left swing phase (from LO to LC), the power of the θ to low β band of the left and right S1 showed opposite changes under FF and FU conditions (a significant ERD appeared in the left S1, and a significant ERS appeared in the right S1). However, a significant ERD appeared in the bilateral S1 under the UF condition. In the last phase of the gait cycle (from LC to RO), a significant ERD appeared in the θ to low β band of the bilateral S1, especially under FU and UF conditions. Interestingly, a significant ERD appeared in the high β (20–30 Hz) band in the left S1 area under all conditions and spanned almost the entire gait cycle, while the right S1 showed opposite changes. In the γ band, a significant ERD appeared in the left S1 under FU and UF conditions and spanned the entire gait cycle, while a significant ERS appeared under the FF condition. In the right S1 area, a significant ERS appeared in the γ band of the entire gait cycle under the FU condition, while a significant ERD appeared in the other two conditions.

The θ band power of the RSP also showed significant changes (Figure 4J and Figure 5E), that is, significant desynchronization in the entire gait cycle under the FU condition, while under the other conditions there was significant synchronization. Interestingly, in the left pre-swing phase and left swing phase (from RC to LC), the α and β band powers under FU and UF conditions showed stronger ERDs compared to the FF condition. Thereafter, the α and β band powers under all conditions rebounded. In the γ band, a significant ERD appeared under FU and UF conditions, while a significant ERS appeared under the FF condition.

### 3.4. Power Spectral Density

**Figure 6:**
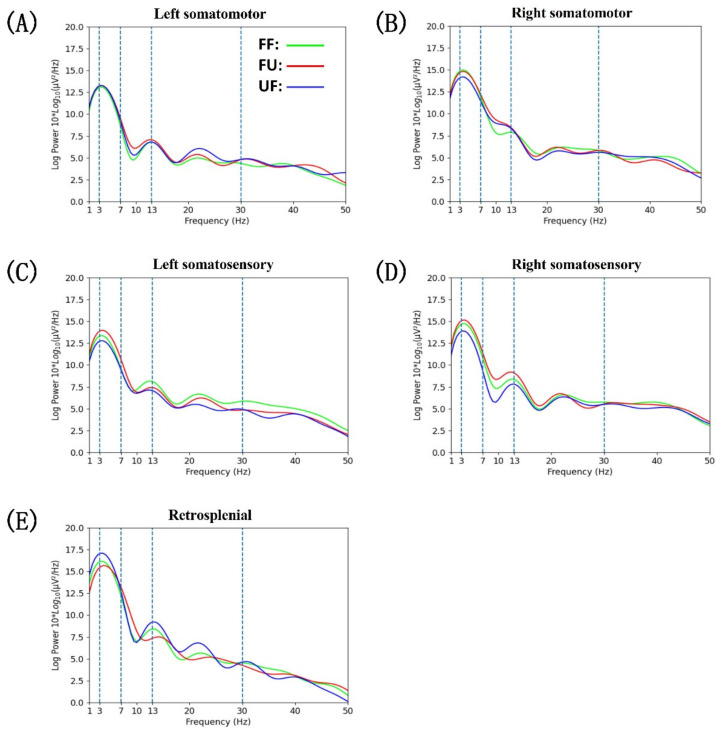
(**A**–**E**) Average power spectral density by cluster for the three experimental walking conditions. (1) FF (green line); (2) FU (red line); and (3) UF (blue line).

Statistical analysis results of the PSD results showed that the difference appeared in the left and right sensory brain areas (Figure 6). In the left somatosensory area (Figure 6C), we found that there were significant differences in the θ band (F(2,252) = 5.400, *p* = 0.005), β band (F(2,252) = 3.821, *p* = 0.023), and the γ band (F(2,252) = 4.510, *p* = 0.012). Post-hoc tests showed that the power in the θ band under the FU condition was greater than that under FF and UF conditions (FF vs. FU: *p* = 0.009; UF vs. FU: *p* = 0.006). In the β band, the power under the UF condition was significantly smaller than that under the FF condition (*p* = 0.01), and the differences between FF and FU conditions were not significant; however, a trend (*p* = 0.057) was evident. In the γ band, compared with the FF condition, the power under FU and UF conditions was significantly decreased (FU vs. FF: *p* = 0.025; UF vs. FF: *p* = 0.007). In the right somatosensory area (Figure 6D), the PSD differences mainly appeared in the θ band (F(2,197) = 3.037, *p* = 0.047) and α band (F(2,197) = 5.069, *p* = 0.007). Post-hoc tests showed that the θ band power under the UF condition was significantly lower than that under FF and UF conditions (UF vs. FF: *p* = 0.05; UF vs. FU: *p* = 0.018), and the same trend appeared in the α band (UF vs. FF: *p* = 0.046; UF vs. FU: *p* = 0.004).

## 4. Discussion

Although previous studies have analyzed the cortical activities of humans and animals during walking, little attention has been paid to how the brain responds to unexpected terrains during this activity. In this study, we used a rat model and designed a series of behavioral tasks, coupled with classic EEG signal processing methods, to analyze the cortical dynamics of rats after encountering an unexpected terrain. Our results showed that the gait and cortical activity of rats after encountering the unexpected terrain were significantly different from normal walking, and varied with the terrain conditions.

Several studies have reported that the motor cortex plays an important role in gait adjustment [44,45,46]. Compared with normal walking, a stronger ERD appeared in the θ band of the left and right M1 after encountering the unexpected terrain (Figure 4B,D and Figure 5A,B). Previous studies have shown that the power of the θ band increases when subjects paid attention to external stimuli, but decreases when subjects paid more attention to tasks than to external stimuli [47,48,49,50,51]. As such, we speculate that rats paid more attention to their gait adjustment in order to minimize the influence of the change in the terrain. The kinematics results showed that after the right hind paw unexpectedly contacted a different terrain, the left hind paw was lifted earlier than in normal walking, and it took less time under the UF condition (Figure 3B). This strategy in rats is consistent with that in cats [52]. In this phase, compared with normal walking, a stronger ERD appeared in the α band of the right M1, and it was the strongest under the UF condition (Figure 4D and Figure 5B). A previous study has demonstrated that the desynchronization of the α band may reflect an increased disposition for motor adjustments [53], and an increase in the desynchronization of the α band may mean that the motor cortex is more involved in movement [54]. In addition, an increase in task difficulty will also make the motor cortex more active [21]. In this study, the desynchronization of the α band may have been due to the fact that the unexpected terrain change increased the difficulty of walking for rats, which made the motor cortex more involved in the movement adjustment. Interestingly, in the left swing phase, the time interval in the FU condition was shorter, while it was longer in the UF condition compared with the other two conditions (Figure 3C). From the results of the locomotion velocity of the left hind limb, the rat was indeed faster under the FU condition and slowed down under the UF condition (Figure 3G). In addition, the gait length under the FU condition in this phase was significantly increased compared to the other two conditions (Figure 3H). These findings consistent with previous research results on humans, which showed that when individuals walk on irregular terrains, they will increase the gait length to minimize the number of contacts with the irregular terrain [55]. These kinematics results showed that the uneven terrain made the posture of the rats unstable, which caused the rats to change their gait pattern. Previous studies have reported that posture instability can lead to an increase in the power of the γ band, and the ERS in the γ band of the motion-related cortex may be related to the coordination of the limbs [56]. Our research yielded similar results; we found that there was a significant ERS in the γ band of the left M1 under the UF condition (Figure 4B). Therefore, the ERS in the γ band likely represented M1 to actively participate in posture adjustment. Interestingly, we noticed that rats stood on the uneven terrain for longer than on the flat terrain before taking the next step (Figure 3D). A previous study demonstrated that when humans walk on an uneven terrain, their stepping time increases [2], and the change in step time is due to the change in the time of the standing phase [57]. The increase in the time of the double-support phase (form LC to RO) may be beneficial to acquire more time to react to the obstacles [58]. It is also related to the fear of falling [4,59]. At the same time, the β band of the bilateral M1 showed stronger desynchronization when rats stood on an uneven terrain (Figure 4B,D and Figure 5A,B). The ERD of β band has been associated with movement preparation [60]. In this study, the stronger desynchronization of the bilateral M1 in the standing phase likely indicated that the cortex was more actively involved in motor planning.

Sensory afferents contribute to the update of gait patterns after sudden perturbations [61], and sensory areas are closely related to sensory processing and play an important role in movement [62,63]. The current study demonstrated that when the rat’s hind paw contacted the ground, a significant θ band ERS appeared on the contralateral S1 (Figure 4F,H). It is worth noting that the power increase in the θ band under the FU condition was more significant than under the other conditions in the left S1 (Figure 5C and Figure 6C). In addition, a significant θ band ERS also appeared in the right S1 when the left hind limb was swinging (from LO to LC), except under the UF condition (Figure 4H and Figure 5D). Previous studies have reported that the increase of power in the θ band is related to changes in the body’s balance state [29,64,65]. Furthermore, the proprioception is closely related to the maintenance of balance [3,29]. A study on cats has demonstrated that muscle afferents of the foot and lower leg generate high frequency bursts when the paw contacted the ground [66]. In addition, hind limb muscles may stretch or compress during limb swings, which may increase proprioceptive input [67,68]. These results seem to indicate that there is a connection between the increase of θ band power and proprioception. In this study, rats were affected by the uneven terrain, which increased the input from the muscles. Therefore, the power synchronization in the θ band may indicate that rats shifted their attention to the processing of proprioceptive information. Interestingly, the power desynchronization of the θ band often appeared on the contralateral S1 in the single support phase or on the bilateral S1 in the double-support phase, and it decreased further after encountering the terrain change (Figure 4F,H and Figure 5C,D). Similar to our results, a previous study has also reported that when humans walk with eyes closed, the θ band power desynchronization appeared in the single support phase [17]. One possible explanation is that when rats cannot perceive terrain changes visually, they will use other methods, such as obtaining sensory information from mechanoreceptors on their paws, to guide movement and maintain a stable posture. Previous studies have also demonstrated that mechanoreceptors play an important role in maintaining posture stability [68,69]. Therefore, the desynchronization of the θ band power was likely a sign that the cortex was more involved in the processing of sensory information caused by the external stimuli. We also noticed that the power changes in the α and low β band are synchronized with the θ band (Figure 4F,H), while the power modulation in the α and β bands of the somatosensory areas were related to the processing of sensory information [16,70]. We speculate that the synchronization in the α and low β bands means that S1 is involved in the processing of proprioceptive information, while the desynchronization means that S1 is involved in the processing of sensory information caused by the external stimuli. One unanticipated finding was that when the right hind paw contacted the ground, the θ band power did not change synchronously with the α to low β power, but the opposite change did occur (Figure 4H). When the left hind limb was swinging, the ERS did not appear in the θ to low β frequency range like the other two conditions, but ERD appeared. From the results of PSD analysis, the θ and α band power was significantly decreased under the UF condition (Figure 6D). These results indicate that even if the rat is left the uneven terrain, the sensory cortex is still actively involved in the processing of sensory information caused by the uneven terrain, which may be caused by the continuous sensory effect. Interestingly, we noticed that the left and right S1 power changes were opposite, especially in the high β band. This is similar to the results of previous studies; when left thumb was stimulated first, the sensitivity of the right thumb decreased [71,72]. Another important finding was that a significant ERD appeared in the γ band of the left S1 after encountering the unexpected terrain changes. The PSD results also showed that the power of the γ band under the conditions of FU and UF was decreased further (Figure 6C). It is likely that the unexpected terrain change increased the difficulty of movement in rats, which made the sensory cortex more involved in the processing of sensory information. Other studies have also reported that as the difficulty of the task increases, there is a significant desynchronization of the power in the γ band [18,73], and the involvement of the cortex also increases [74]. However, under the FU condition, the γ band of the right S1 showed a significant ERS, which was opposite to those of the other conditions (Figure 4H and Figure 5D). Similar to our results, a previous study has demonstrated that the synchronization of the sensorimotor and posterior parietal cortex increases when humans perform more complex movements [54]. Therefore, we speculate that the γ band ERS may be caused by the S1 integrated sensory information, which is used to guide the adjustment of left hind limb and to adapt to the uneven terrain.

Significant changes also appeared in the RSP (Figure 4J and Figure 5E). When rats contacted the uneven terrain, the θ band power was significantly desynchronized, while the opposite was true when they contacted the flat ground. The θ band activity is related to working memory function [75]. Furthermore, rats can recognize the characteristics of objects only by touch [76], and RSP activity increases during object recognition [77]. In this study, the desynchronization of the θ band after encountering the uneven terrain may indicate that the cortex was actively involved in the recognition of complex terrain features. Compared with the uneven terrain, rats seem to be more experienced in responding to the flat terrain, which not only reduces the extent of cortical involvement but also allows rats to respond faster. A previous study reported that humans can respond more quickly to unexpected terrain changes under the guidance of previous experiences [1]. This is consistent with our kinematics results, that is, after encountering an unexpected terrain, if the terrain in front was flat, the reaction time of the rat was shorter (Figure 3B). Compared with normal walking, the α and β band powers were significantly decreased after the rat encountered the unexpected terrain until it completely transitioned to another terrain (Figure 5E), and the power rebound after the transition was completed. The ERD in the α and β bands may indicate that the cortex is actively involved in cognitive information processing [78,79]. The RSP of rats is more active when new terrain information was acquired [80]. Therefore, the ERD in the α and β bands may indicate that the RSP is actively involved in the processing of new terrain information, while the ERS is the signal of completion of the information processing. Additionally, the RSP plays an important role in the integration of multiple sensory information and route planning [77,80]. Previous studies have demonstrated that power modulation in the γ band is related to the integration of multiple sensations [16,54,81]. We also observed that after encountering the unexpected terrain, the γ (30–40 Hz) band power of almost the entire gait cycle showed significant desynchronization (Figure 5E). Therefore, the ERD in the γ band of the RSP may indicate that the cortex is more involved in the integration of sensory information after terrain changes for route planning.

Due to the difficulty of surgery and the long training cycle of rats, our data came from limited subjects, which may hamper the possibility of generalization. Increasing the number of subjects can get more reliable conclusions. In this study, our definition of unexpected terrain was limited to uneven terrain, and other unexpected terrains, such as unexpected slippery surface or unexpected loss of ground support, were not discussed, which made our conclusions lack of generality. Therefore, it was necessary to analyze the behavior and cortical activity of rats when they encounter different types of unexpected terrain, so as to obtain a general conclusion. In addition, we did not discuss whether the lack of environmental cues affects the normal walking of rats, and the strategies for bipedal-walking rats with blocked vision to cope with different unexpected terrains. Further research tackling these issues was warranted. Finally, our conclusions were drawn from analyses of EEG signals at the whole brain level. Complementary analyses on the extracellular recording of single unit activity should be further studied the cortical activity after encountering the unexpected terrain.

## 5. Conclusions

Our research investigated the limb kinematics and cortical dynamics of bipedal-walking rats after encountering an unexpected terrain. After encountering the unexpected terrain, the bilateral M1 was more involved in movement adjustment, and played an important role in gait planning and limb coordination, as indicated by the broadband power decrease of θ, α, and β rhythms and a significant γ band power increase. Additionally, the bilateral S1 was more involved in the processing of sensory information and more sensitive to sensory afferent after the terrain change, as evidence by the power modulation of the θ to low β band and γ band. The RSP may be involved in terrain recognition, as indicated by the opposite change of the θ band power after the rats contacted the uneven terrain and flat terrain. Furthermore, the RSP is more active in terrain information update and multi-sensory information integration after the rats encountered the unexpected terrain, as indicated by the stronger power decrease of the α to γ band. These results indicate unexpected terrain-induced changes in cortical activity. This study extends our understanding of the impact of unexpected terrains on cortical activity.

## Figures and Tables

**Table 1:** Hind limbs kinematics data (mean ± SEM) of each gait phase.

		Right Swing Phase	Left Pre-Swing Phase	Left Swing Phase	Right Pre-Swing Phase
Time (s)	FF	0.1774 ± 0.0033	0.1782 ± 0.0053	0.2153 ± 0.0043	1.0513 ± 0.0183
	FU	0.1723 ± 0.0047	0.1603 ± 0.0058	0.1964 ± 0.0036	1.1294 ± 0.0187
	UF	0.1733 ± 0.0044	0.1410 ± 0.0056	0.2345 ± 0.0051	1.0508 ± 0.0210
Gait length (cm)	FF	8.31 ± 0.11		7.79 ± 0.19	
	FU	8.17 ± 0.12		8.39 ± 0.15	
	UF	8.12 ± 0.14		7.71 ± 0.22	
Locomotion velocity (cm/s)	FF	45.78 ± 1.03		39.59 ± 1.39	
	FU	47.05 ± 1.14		44.20 ± 1.64	
	UF	47.88 ± 1.07		35.33 ± 1.53	

**Table 2:** IC clusters and cluster centroid locations.

Cluster	Talairach Coordinates	Cortical Location	Number of Subjects and ICs
Left somatomotor	−26, 28, 52	Left primary motor area	4Ss, 8ICs
Right somatomotor	24, 26, 51	Right primary motor area	3Ss, 9ICs
Left somatosensory	−32, −5, 60	Left primary somatosensory area	4Ss, 12ICs
Right somatosensory	27, −7, 64	Right primary somatosensory area	3Ss, 11ICs
Retrosplenial	−3, −46, 62	Retrosplenial area	4Ss, 14ICs

## Data Availability

The datasets obtained during the current study are available from the corresponding author on reasonable request.
